# Successful Combined Autologous Blood and Minocycline Pleurodesis for Intractable Bilateral Pneumothorax in an Elderly Patient With Combined Pulmonary Fibrosis and Emphysema: A Case Report

**DOI:** 10.1002/rcr2.70358

**Published:** 2025-09-22

**Authors:** Eitetsu Koh

**Affiliations:** ^1^ Department of Thoracic Surgery Tokyo Women's Medical University, Yachiyo Medical Center Yachiyo Chiba Japan

**Keywords:** autologous blood, combined pulmonary fibrosis and emphysema, elderly, minocycline, pleurodesis, pneumothorax

## Abstract

Bilateral pneumothorax in elderly patients with combined pulmonary fibrosis and emphysema (CPFE) is rare and often precludes surgery. We report an 86‐year‐old man with CPFE and a persistent air leak unresponsive to tube drainage. Combined pleurodesis via the chest tube using 50 mL autologous blood and 100 mg minocycline diluted in 50 mL saline achieved cessation after two sessions 3 days apart. Two months later, contralateral pneumothorax occurred and was successfully treated using the same protocol. No procedure‐related complications were observed; no recurrence was seen at 6‐month follow‐up. This simple bedside approach may be a practical option when surgery is contraindicated.

## Graphical abstract

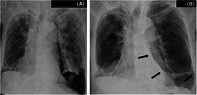

## 1 Introduction

Combined pulmonary fibrosis and emphysema (CPFE) presents unique therapeutic challenges due to parenchymal destruction, poor lung compliance, and comorbidities. In this setting, spontaneous pneumothorax may be refractory to drainage, and operative options are often unsuitable in the very elderly. Autologous blood patch and minocycline pleurodesis have each shown efficacy for persistent air leak [1, 2, 3, 4], but their combined use in CPFE is seldom reported. We describe reproducible, complication‐free control of bilateral pneumothoraces in an octogenarian with CPFE using combined autologous blood and minocycline pleurodesis.

In frail patients with CPFE, prolonged air leak is associated with morbidity and a longer hospital stay. After 5 days of persistent air leak and given the patient's very high operative risk, we elected to perform combined pleurodesis at the first attempt to maximise the likelihood of early air‐leak closure and to minimise chest‐tube dwell time. Autologous blood provides a mechanical seal and promotes fibrin adhesion [1, 2, 3, 5], whereas minocycline induces pleural inflammation and fibrosis [4]. Using both agents together was considered reasonable in this context, acknowledging the limited published data on combined therapy.

## 2 Case Report

**FIGURE 1:**
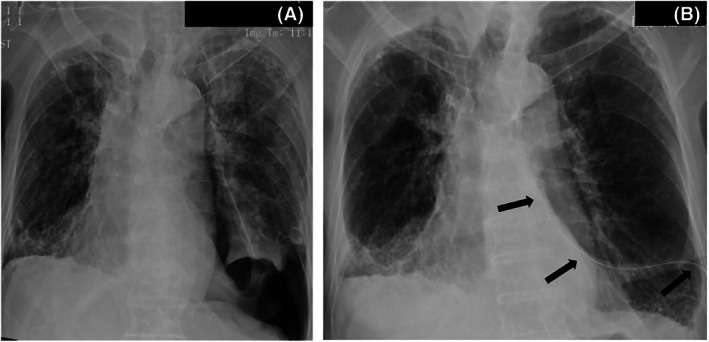
(A) Chest radiograph showing a left pneumothorax. (B) Post–chest tube radiograph demonstrating tube position (arrows) and partial re‐expansion.

An 86‐year‐old man with a 60 pack‐year smoking history and established CPFE presented with acute dyspnea. Chest radiography showed a left‐sided spontaneous pneumothorax (Figure 1A). A chest tube was placed; however, a persistent air leak remained after 5 days despite adequate drainage (Figure 1B). Given advanced age and markedly impaired pulmonary reserve, surgery was deemed inappropriate.

Under water‐seal drainage, we instilled 50 mL of autologous venous blood followed by 100 mg of minocycline dissolved in 50 mL of normal saline via the chest tube, following published approaches to intrapleural autologous blood instillation [2, 3]. The tube was briefly elevated to prevent immediate egress. Air leak decreased but persisted; a second, identical session on Day 3 led to complete cessation. Lung re‐expansion was confirmed radiographically, and the tube was removed.

**FIGURE 2:**
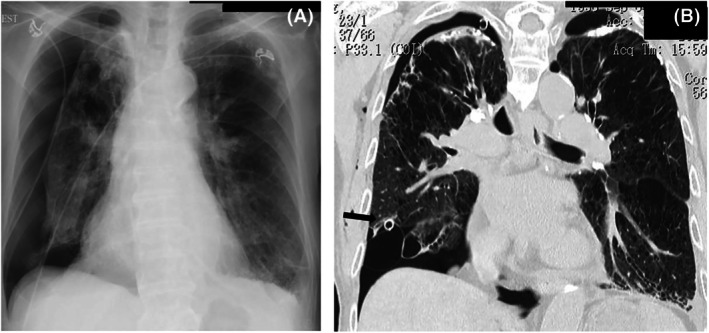
(A) Chest radiograph showing a right pneumothorax. (B) Coronal chest CT image demonstrating upper‐lobe emphysema and lower‐lobe fibrosis consistent with CPFE; chest tube in situ (arrow) with partial re‐expansion.

Two months later, he developed a right‐sided spontaneous pneumothorax (Figure 2A). Computed tomography demonstrated a CPFE pattern with upper‐lobe–predominant emphysema and lower‐lobe fibrosis (Figure 2B). Using the same two‐session protocol on the right side resulted in durable air‐leak cessation and full re‐expansion. No fever, hypotension, empyema, or pain requiring escalation occurred. The patient was discharged home and remained recurrence‐free at 6 months.

## 3 Discussion

Autologous blood functions as a mechanical sealant and promotes fibrin‐mediated adhesion [1, 2]. Minocycline induces pleural inflammation and fibrosis and has randomised‐trial evidence for efficacy in spontaneous pneumothorax [4]. Individually, these methods show high success rates for persistent air leak [1, 2, 3, 4]; combining them may confer synergistic efficacy while maintaining a favourable safety profile. This case demonstrates the feasibility and reproducibility of combined therapy on both hemithoraces in a frail, nonsurgical CPFE patient without infectious or systemic complications. Operative bullectomy may be precluded by poor reserve in such patients, whereas this approach is low‐cost, executable at the bedside, and globally accessible. Prospective evaluation is warranted to clarify indications, dosing (e.g., blood volume 50–100 mL; minocycline 100 mg), and optimal timing in secondary spontaneous pneumothorax with underlying interstitial disease.

Why we chose upfront combination rather than step‐up single‐agent therapy. In non‐surgical candidates with interstitial lung disease, a step‐up strategy (single agent then adding a second agent upon failure) may prolong chest‐tube duration and the risks associated with persistent air leak. Our intent was to expedite closure by combining a mechanical seal (autologous blood) with chemical pleurodesis (minocycline) from the outset. We recognise that either agent alone might have sufficed; this uncertainty is now stated explicitly as a limitation. Future comparative studies should test whether upfront combination reduces time to air‐leak cessation versus sequential single‐agent approaches.

A key limitation is that we cannot exclude that either agent alone would have been effective; our choice of upfront combination was guided by the patient's frailty and the clinical priority to avoid prolonged air leak.

In conclusion, combined autologous blood and minocycline pleurodesis is a safe, effective, and resource‐sparing option for intractable bilateral pneumothorax in elderly CPFE patients who are not candidates for surgery.

## Ethics Statement

The Institutional Review Board of Tokyo Women's Medical University Yachiyo Medical Center reviewed and approved this report (approval no. 5671). The treatment described was part of routine clinical care; no investigational drugs, devices, or randomisation were used.

## Consent

The author declare that written informed consent was obtained for the publication of this manuscript and accompanying images and attest that the form used to obtain consent from the patient complies with the Journal requirements as outlined in the author guidelines.

## Conflicts of Interest

The author declares no conflicts of interest.

## Data Availability

The data that support the findings of this study are available from the corresponding author upon reasonable request.

## References

[1] R. Dumire , M. M. Crabbe , F. G. Mappin , and L. J. Fontenelle , “Autologous “Blood Patch” Pleurodesis for Persistent Pulmonary Air Leak,” Chest 101, no. 1 (1992): 64–66, 10.1378/chest.101.1.64. 1729112

[2] H. S. Lee , H. Y. Kim , J. Lee , et al., “Intrapleural Instillation of Autologous Blood for Persistent Air Leak in Spontaneous Pneumothorax,” Respirology 12, no. 5 (2007): 719–723, 10.1111/j.1440-1843.2007.01125.x.17875061

[3] G. Q. Cao , J. Kang , F. Wang , and H. Wang , “Intrapleural Instillation of Autologous Blood for Persistent Air Leak in Spontaneous Pneumothorax in Patients With Advanced Chronic Obstructive Pulmonary Disease,” Annals of Thoracic Surgery 93, no. 5 (2012): 1652–1657, 10.1016/j.athoracsur.2012.01.093. 22459543

[4] J. S. Chen , K. T. Tsai , H. H. Hsu , et al., “Simple Aspiration and Drainage and Intrapleural Minocycline Pleurodesis Versus Simple Aspiration and Drainage for the Initial Treatment of Primary Spontaneous Pneumothorax: A Randomized Controlled Trial,” Lancet 381, no. 9874 (2013): 1277–1282, 10.1016/S0140-6736(12)62170-9. 23489754

[5] K. Aihara , T. Handa , S. Nagai , et al., “Efficacy of Blood‐Patch Pleurodesis for Secondary Spontaneous Pneumothorax in Interstitial Lung Disease,” Internal Medicine 50, no. 11 (2011): 1157–1162, 10.2169/internalmedicine.50.4645. 21628929

